# Supplementary material for: Evaluation of pragmatic oxygenation measurement as a proxy for Covid-19 severity
Source: Nat Commun. 2023 Nov 15;14:7374. doi: 10.1038/s41467-023-42205-6 (PMC10651917; doi:10.1038/s41467-023-42205-6)
Supplement: Supplementary file 1 — Supplementary Information [file 41467_2023_42205_MOESM1_ESM.pdf]

Supplementary information

Supplementary Methods

Effect size estimation

This section details how effect sizes associated with a given treatment effect were calculated for different outcome measures.

S/F_{94} and sustained WHO ordinal scale improvement

We used a logistic model with mortality as the dependent variable, and separately estimated relationships with S/F_{94} and WHO ordinal scale improvement,

$$\ln\left(\frac{p_i}{1-p_i}\right) = \beta_0 + \beta_1 y_i + \boldsymbol{\beta} \cdot z_i. \tag{1}$$

Here, p_i is the probability of day 28 mortality for individual i, β_0 and β_1 are scalar coefficients, $\boldsymbol{\beta}$ is an n-dimensional vector of coefficients, y_i is the outcome measure (S/F_{94} or sustained WHO ordinal scale improvement encoded as a binary variable taking values 0 or 1), and z_i is an n-dimensional vector of covariates.

Let $\hat{\beta}_0, \hat{\beta}_1, \hat{\boldsymbol{\beta}}$ be consistent estimators for $\beta_0, \beta_1, \boldsymbol{\beta}$ respectively. $\hat{p_i}$ is defined as the corresponding predicted probability of day 28 mortality for patient i,

$$\ln\left(\frac{\hat{p}_i}{1-\hat{p}_i}\right) = \hat{\beta}_0 + \hat{\beta}_1 y_i + \hat{\boldsymbol{\beta}} \cdot z_i. \tag{2}$$

We consider an individual-specific treatment effect given by

$$p_i \to f(p_i) \tag{3}$$

for some function f taking values in $[0,1]$, that may in principle depend on all covariates. The changes $\Delta_i, \hat{\Delta}_i$ in the outcome measure associated with this treatment effect for individual i keeping all other covariates constant in Equation 1 and Equation 2 satisfy

$$\ln\left(\frac{f(p_i)}{1-f(p_i)}\right) = \beta_0 + \beta_1(y_i + \Delta_i) + \boldsymbol{\beta}\cdot z_i. \tag{4}$$

$$\ln\left(\frac{f(\hat{p_i})}{1-f(\hat{p_i})}\right) = \hat{\beta}_0 + \hat{\beta}_1(y_i + \hat{\Delta}_i) + \hat{\boldsymbol{\beta}}\cdot z_i. \tag{5}$$

Our estimator for the expected change in y_i associated with the treatment effect f is obtained by subtracting Equation 2 from Equation 5, dividing by $\hat{\beta}_1$ and averaging over the sample,

$$\hat{\mu}_\Delta := \frac{1}{\hat{\beta}_1 I}\sum_{i=1}^{I}\ln\left(\frac{f(\hat{p_i})(1-\hat{p}_i)}{\hat{p}_i(1-f(\hat{p_i}))}\right). \tag{6}$$

This is a consistent estimator for $\mu_\Delta = \mathbb{E}[\Delta_i]$, which follows from the fact that $\hat{\beta}_0, \hat{\beta}_1, \hat{\boldsymbol{\beta}}$ are consistent estimators for $\beta_0, \beta_1, \boldsymbol{\beta}$.

In the results presented in this paper, a treatment effect that is a relative risk reduction of e.g. 15% means that f is defined by

$$f(x) = 0.85x. \tag{7}$$

That is, the patient's predicted risk of mortality is multiplied by 0.85.

Protocolised S/F_{94}

In the ISARIC dataset, S/F_{94} measurements are made opportunistically. We expect protocolised S/F_{94} measurements to be more precise and to be a better predictor of mortality that opportunistic measurements. This in turn implies that smaller sample sizes will be required when using protocolised S/F_{94} measurements as an intermediate endpoint in clinical trials.

In order to calculate the magnitude of this effect, we assume a measurement error model of the following form

$$y_i = \alpha_0 + \alpha_1 x_i + \epsilon_i, \tag{8}$$

where x_i is a protocolised measurement of S/F_{94}, α_0, α_1 are scalar coefficients, and ϵ_i is a residual.

We will use the notation

$$\mu_x := \mathbb{E}[x_i] \tag{9}$$

$$\mu_y := \mathbb{E}[y_i] \tag{10}$$

$$\sigma_x^2 := \mathrm{Var}[x_i] \tag{11}$$

$$\sigma_y^2 := \mathrm{Var}[y_i]. \tag{12}$$

We also assume the moment conditions

$$\mathbb{E}[\epsilon_i] = 0 \tag{13}$$

$$\mathbb{E}[\epsilon_i x_i] = 0. \tag{14}$$

Combining Equation 13 and Equation 14 with Equation 8 gives

$$\alpha_0 = \mu_y - \frac{\rho_{xy}\sigma_y\mu_x}{\sigma_x} \tag{15}$$

$$\alpha_1 = \frac{\rho_{xy}\sigma_y}{\sigma_x} \tag{16}$$

where ρ_{xy} is the Pearson correlation between x_i and y_i.

Combining Equation 1 with Equation 8 gives

$$\ln\left(\frac{p_i}{1-p_i}\right) = \beta_0' + \beta_1' x_i + \beta_1 \epsilon_i + \boldsymbol{\beta} \cdot z_i, \tag{17}$$

where $\beta_0' := \beta_0 + \alpha_0\beta_1$ and $\beta_1' = \alpha_1\beta_1$.

Assuming α_0, α_1 are the same on day 0 and day 5, it is possible to show that

$$\rho_{xy}^2 = \frac{\rho_{y(5)y(0)}}{\rho_{x(5)x(0)}}, \tag{18}$$

where $\rho_{y(5)y(0)}$ is the Pearson correlation between opportunistic S/F_{94} measurements on day 5 and day 0, and $\rho_{x(5)x(0)}$ is the Pearson correlation between protocolised S/F_{94} measurements on day 5 and day 0. Therefore, α_0 and α_1 can be consistently estimated by using consistent estimators for μ_x, μ_y, σ_x, σ_y, $\rho_{x(5)x(0)}$ and $\rho_{y(5)y(0)}$ together with Equation 15, Equation 16 and Equation 18. Denoting these estimators $\hat{\alpha}_0$ and $\hat{\alpha}_1$ respectively, we define

$$\ln\left(\frac{\hat{p}_i}{1-\hat{p}_i}\right) := \hat{\beta}_0' + \hat{\beta}_1' x_i + \hat{\boldsymbol{\beta}} \cdot z_i, \tag{19}$$

where $\hat{\beta}_0' := \hat{\beta}_0 + \hat{\alpha}_0\hat{\beta}_1$, $\hat{\beta}_1' = \hat{\alpha}_1\hat{\beta}_1$, and $\hat{p}_i$ is the corresponding estimate of the probability of day 28 mortality for individual i. We have

$$\hat{\beta}_0' \xrightarrow{p} \beta_0' \tag{20}$$

$$\hat{\beta}_1' \xrightarrow{p} \beta_1', \tag{21}$$

where $\xrightarrow{p}$ denotes convergence in probability. If in addition we assume $\{\epsilon_i\}_{i=1}^{I}$ is mean ergodic, then using Equation 13 gives

$$\text{plim}_{I\to\infty}\frac{1}{I}\sum_{i=1}^{I}\left[\ln\left(\frac{p_i}{1-p_i}\right)-\ln\left(\frac{\hat{p}_i}{1-\hat{p}_i}\right)\right]=0. \tag{22}$$

Now consider an individual-specific treatment effect given by

$$p_i \to f(p_i) \tag{23}$$

for some function f taking values in $[0,1]$, that may in principle depend on all covariates. Let $\Delta_i, \hat{\Delta}_i$ be the change in x_i associated with this treatment effect in Equation 17 and Equation 19 respectively. That is,

$$\ln\left(\frac{f(p_i)}{1-f(p_i)}\right)=\beta_0'+\beta_1'(x_i+\Delta_i)+\beta_1\epsilon_i+\boldsymbol{\beta}\cdot z_i. \tag{24}$$

$$\ln\left(\frac{f(\hat{p}_i)}{1-f(\hat{p}_i)}\right)=\hat{\beta}_0'+\hat{\beta}_1'(x_i+\hat{\Delta}_i)+\hat{\boldsymbol{\beta}}\cdot z_i. \tag{25}$$

Our estimator for the average change in protocolised S/F_{94} associated with the treatment effect f is obtained by subtracting Equation 19 from Equation 25, dividing by $\hat{\beta}_1'$ and averaging over the sample,

$$\hat{\mu}_\Delta=\frac{1}{\hat{\beta}_1' I}\sum_{i=1}^{I}\ln\left(\frac{f(\hat{p}_i)(1-\hat{p}_i)}{\hat{p}_i(1-f(\hat{p}_i))}\right). \tag{26}$$

We now show this is a consistent estimator for $\mu_\Delta := \mathbb{E}[\Delta_i]$ at first order in ϵ_i.

Consider

$$\hat{\mu}_\Delta-\frac{1}{I}\sum_{i=1}^{I}\Delta_i=\frac{1}{I}\sum_{i=1}^{I}\left(\frac{1}{\hat{\beta}_1'}\left[\ln\left(\frac{f(\hat{p}_i)}{1-f(\hat{p}_i)}\right)-\ln\left(\frac{\hat{p}_i}{1-\hat{p}_i}\right)\right]-\frac{1}{\beta_1'}\left[\ln\left(\frac{f(p_i)}{1-f(p_i)}\right)+\ln\left(\frac{p_i}{1-p_i}\right)\right]\right) \tag{27}$$

We assume f is analytic, and Taylor expand the third term in Equation 27 to first order around $\epsilon_i=0$. Let $g(\epsilon_i)=\ln\left[\frac{f(p_i)}{1-f(p_i)}\right]$, where p_i is understood to be a function of ϵ_i via Equation 17. Then

$$\hat{\mu}_{\Delta} - \frac{1}{I}\sum_{i=1}^{I}\Delta_i \simeq \frac{1}{I}\sum_{i=1}^{I}\Bigg(\frac{1}{\hat{\beta}'_1}\left[\ln\left(\frac{f(\hat{p}_i)}{1-f(\hat{p}_i)}\right) - \ln\left(\frac{\hat{p}_i}{1-\hat{p}_i}\right)\right] - \frac{1}{\beta'_1}\left[g(\epsilon_i = 0) + g'(\epsilon_i = 0)\epsilon_i + \ln\left(\frac{p_i}{1-p_i}\right)\right]\Bigg) \tag{28}$$

Now taking probability limits and using Equation 21, Equation 22, as well as

$$\text{plim}_{I\to\infty}\left(g(\epsilon_i = 0) - \ln\left(\frac{f(\hat{p}_i)}{1-f(\hat{p}_i)}\right)\right) = 0 \tag{29}$$

$$\text{plim}_{I\to\infty}\sum_{i=1}^{I}\epsilon_i = 0, \tag{30}$$

we obtain

$$\text{plim}_{I\to\infty}\left(\hat{\mu}_{\Delta} - \mu_{\Delta}\right) = 0. \tag{31}$$

This is the desired consistency result.

We calculated effect sizes with protocolised S/F_{94} as outcome measure, with μ_x, σ_x, $\rho_{x(5)x(0)}$ estimated from the ISARIC4C data. We took our estimate of μ_y to be equal to our estimate of μ_x. σ_y and $\rho_{y(5)y(0)}$ were estimated using data from the RECOVERY trial.

WHO ordinal scale

We used a proportional odds model to estimate the relationship between day 28 WHO ordinal scale category and individual characteristics,

$$\ln\left(\frac{p_{ij}}{1-p_{ij}}\right) = \beta_{0j} + \boldsymbol{\beta}\cdot z_i. \tag{32}$$

Here, p_{ij} is the probability that the WHO ordinal scale category of individual i at day 28 is $\leq j$, β_{0j} are a set of constant terms for each WHO ordinal scale category, β is an n-dimensional vector of coefficients, and z_i is an n-dimensional vector of covariates.

$j = 10$ is the highest WHO ordinal scale category and corresponds to death. Then $q_{i10} := 1 - p_{i10}$ is the probability of day 28 mortality for patient i. We consider an individual-specific treatment effect given by

$$q_{i10} \to f(q_{i10}) \tag{33}$$

for some function f taking values in $[0, 1]$, that may in principle depend on all covariates.

Let $\hat{q}_{i10}$ denote the predicted probability of day 28 mortality for individual i derived from the model Equation 32. Then the rate of predicted day 28 mortality in the sample can be estimated by $\bar{q}_{10} := \frac{1}{I}\sum_{i=1}^{I}\hat{q}_{i10}$. The odds ratio associated with the treatment effect f is estimated by

$$\frac{\left(\frac{f(\bar{q}_{10})}{1-f(\bar{q}_{10})}\right)}{\left(\frac{\bar{q}_{10}}{1-\bar{q}_{10}}\right)} = \frac{f(\bar{q}_{10})\,(1-\bar{q}_{10})}{\bar{q}_{10}[1-f(\bar{q}_{10})]}. \tag{34}$$

We take this as our estimate of effect size.

Quantifying uncertainty

The required sample size and 95% confidence interval when the outcome measure is 1- or 2-level sustained improvement on the WHO scale is noticeably larger than for the other outcome measures, in particular S/F_{94}. There are several reasons for this. The first is that the magnitude of effect size compared to variance of outcome measure is much smaller when 1- or 2-level sustained improvement on the WHO scale is the outcome measure compared to when S/F_{94} is the outcome measure. The second reason is that S/F_{94} is a continuous variable, whereas 1- or 2-level sustained improvement on the WHO scale are Bernoulli variables whose mean is the proportion of people who had a 1 or 2-level sustained improvement on the WHO scale, respectively. The sample size calculation with S/F_{94} as outcome measure relies on a two-sample t-test for testing the hypothesis that the means of two normal distributions with the same variance are equal. In the case of 1- or 2-level sustained improvement on the WHO scale, the procedure is similar except the variances of the two distributions are not equal. The mean of a large number of independent Bernoulli variables with mean μ is approximately normally distributed with mean μ and asymptotic variance $\frac{\mu(1-\mu)}{n}$. Therefore, two sets of Bernoulli distributed variables with different means also have different variances. If we use μ_1 to denote the mean for the control group, and μ_2 to denote the mean for the treatment group, then while $\mu_1 < \mu_2 \leq 0.5$, the variance of the sample mean of the outcome measure increases as the effect size $\mu_2 - \mu_1$ increases. Thus while these conditions hold, and with all else equal, this has the consequence that an outcome measure that is Bernoulli distributed will require larger sample sizes than an outcome measure that has a continuous distribution.

Generation of synthetic data

A synthetic dataset was generated for use in the online sample size calculation tool. This dataset was created using imputation by chained equations in multiple stages. First, empty records for one thousand new patients at day zero were added to the real data. Next, variables that can be reasonably expected to be constant over a 28 day time period (e.g comorbidities such as obesity, dementia, liver disease etc) were imputed by chained equations. New rows were added for day 5 and day 8 for each simulated record, with the constant variables imputed in the

previous step repeated. After this, there were multiple rounds of partitioning the full dataset, containing both real and simulated records, and carrying out imputation by chained equations separately within each piece. For example, a binary variable indicating whether an individual died was imputed, then the full dataset was split into those who died and those who didn't, and a day of death was imputed for the former. Similarly, binary variables indicating whether or not the individual had a one/two level sustained improvement in WHO ordinal severity score were imputed, and used to partition the full dataset. Then the minimum time taken for patients to have a sustained one/two level improvement was imputed amongst those that had this event occur. Appropriate care was taken in order to respect variable bounds, interdependence and the time series structure of the data. For example, if an individual had a sustained two level improvement, they necessarily must also have had a sustained one level improvement, and the time taken for the former to occur must necessarily be greater than or equal to the time taken for the latter to occur. In order to handle this appropriately, we first imputed the time taken for a sustained one level improvement, and after appropriate partitioning, we then imputed the difference in time taken for a sustained one and two level improvement using predictive mean matching. This guaranteed a positive difference, as required.

In each imputation by chained equations, predictive mean matching was used for continuous variables and logistic or multinomial logistic regression was used for categorical variables.

Supplementary Notes

Physiology

Evaluation in ISARIC data

Respiratory Rate

There is large variation in respiratory rate, which is independent of S/F_{94}. As expected, there is a tendency of higher respiratory rates for patients with a lower S/F_{94}. (Supplementary Figure 3)

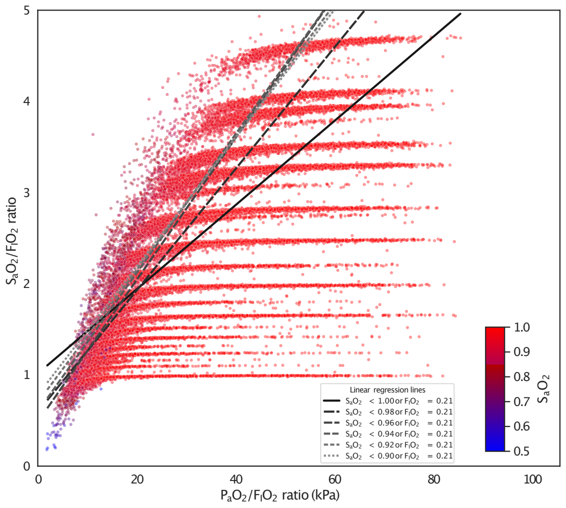

(a) S/F (all values)

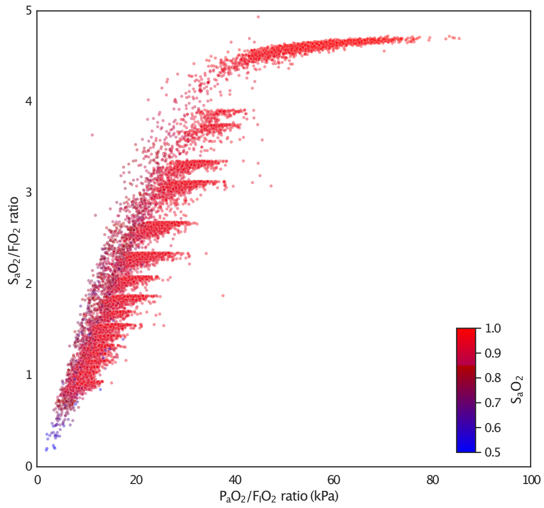

(b) S/F_{94}

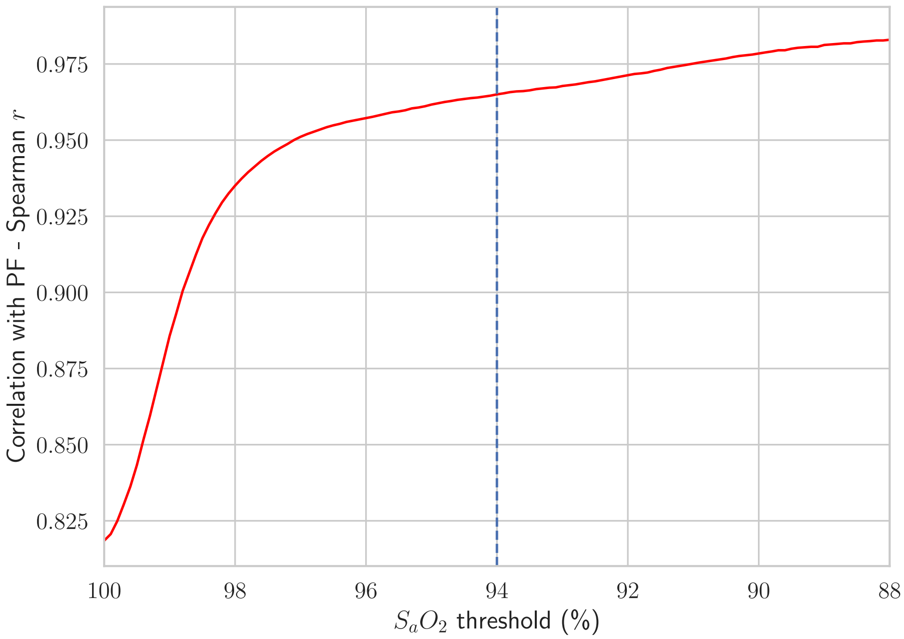

(c) Optimisation by correlation with P/F

Supplementary Figure 1: (a,b) Scatter plots showing real blood gas results from critically ill patients. S/F is plotted against our reference standard, P/F. Points are coloured according the S_aO_2 as shown in the colour scale. (a) including all values with no maximum S_aO_2. Linear regression lines are shown for S/F against P/F in using different maxima for S_aO_2; patients breathing air (F_IO_2=21%) were included in all regression analyses regardless of S_aO_2. (b) shows only values meeting the criteria for S/F_{94}: $S_pO_2 \leq 0.94$ or $F_IO_2 = 21\%$ (c) Optimisation of cut-off value for S_aO_2 using change in correlation coefficient (Spearman r) as the threshold for inclusion is lowered from S_aO_2< 100% to S_aO_2< 80%.

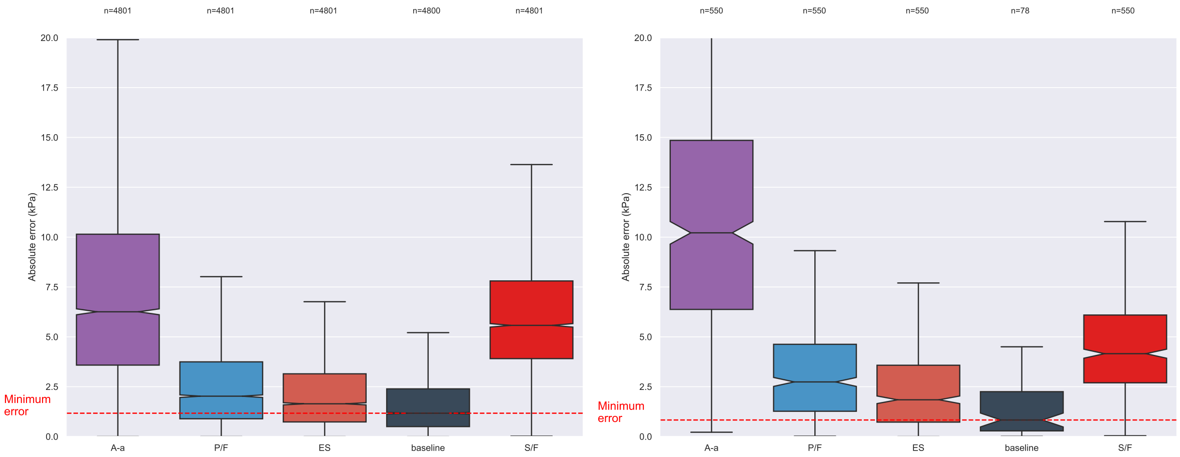

(a) Including all recordings

(b) Restricted to recordings where $S_aO_2 < 0.94$

Supplementary Figure 2: Predictive validity (ability to predict a future P_aO_2 after a change in F_IO_2; absolute error in kPa) of Alveolar- arterial (A-a), P/F ratio (P/F), effective shunt fraction (ES), and S/F ratio (S/F), compared to a baseline (the minimum achievable error, measured as the change in P_aO_2 between pairs of arterial blood gas measurements meeting the other selection criteria, with no change in F_IO_2). Lower values indicate superior validity. Centre line represents median values, box limits represent upper and lower quartiles, whiskers represent minimum and maximum values. All values are included in (a); in (b) only values in which $S_aO_2 < 0.94$ in the first measurement.

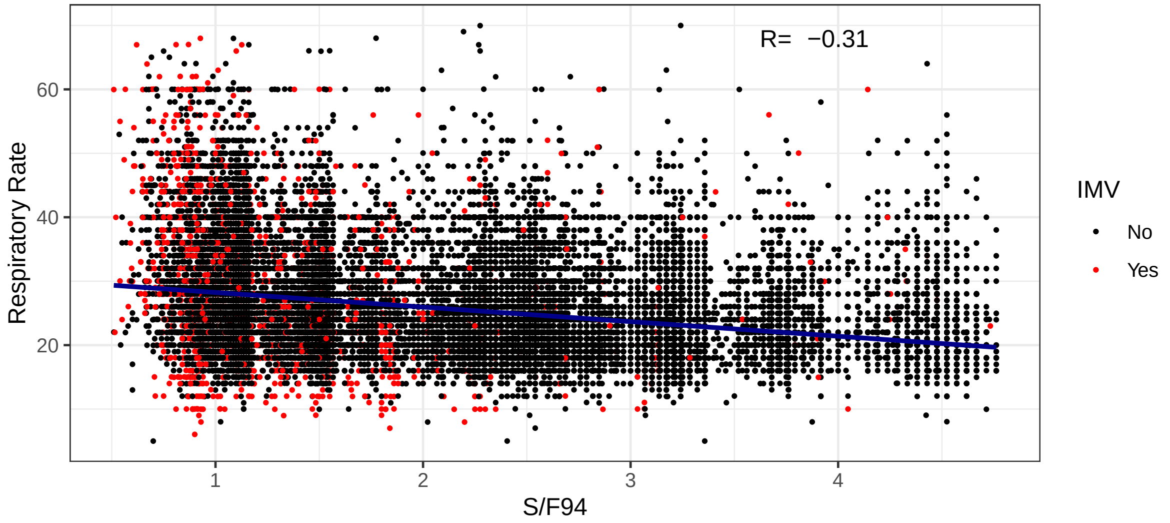

Supplementary Figure 3: Relationship between respiratory rate and the S/F_{94} in 49,727 patients meeting inclusion criteria (age 20-75; oxygen therapy in first 3 days of admission). Patients who received invasive mechanical ventilation at the time of the respiratory rate and S/F_{94} measurement are highlighted in red.

Association between mortality at day 28 and S/F_{94}

The relationship between S/F_{94} on day 0 and mortality in the multivariable model appears counter-intuitive (Supplementary Figure 4a). A univariable regression model with mortality on day 28 as the outcome variable and S/F_{94} on day 0 as the predictor, shows a relationship as expected: there is an increase in mortality risk with a decrease in S/F_{94} on day 0 (Supplementary Figure 4b). Hence the reversed direction in the multivariable model is explained by the effect of change in S/F_{94} between day 0 and day 5, suggesting that patients who are admitted to hospital with moderate oxygenation defects, and deteriorate rapidly, have a slightly increased risk of mortality.

Sensitivity analysis

We carried out a sensitivty analysis not including assigned minimum and maximum values for those who died or were discharged. The logistic regression analysis used to evaluate the association between 28-day mortality and S/F_{94} on day 5, corrected for S/F_{94} on day 0, showed an OR of 0.33. For a 1 unit increase in S/F_{94}, the odds of 28-day mortality decrease by 66%. The mean of S/F_{94} decreased from 2.39 in the main analysis to 2.17 in this sensitivity analysis, which was due to the fact that a larger number of patients were discharged than died. The standard deviation of S/F_{94} was also smaller in this sensitivity analysis (1.00, compared

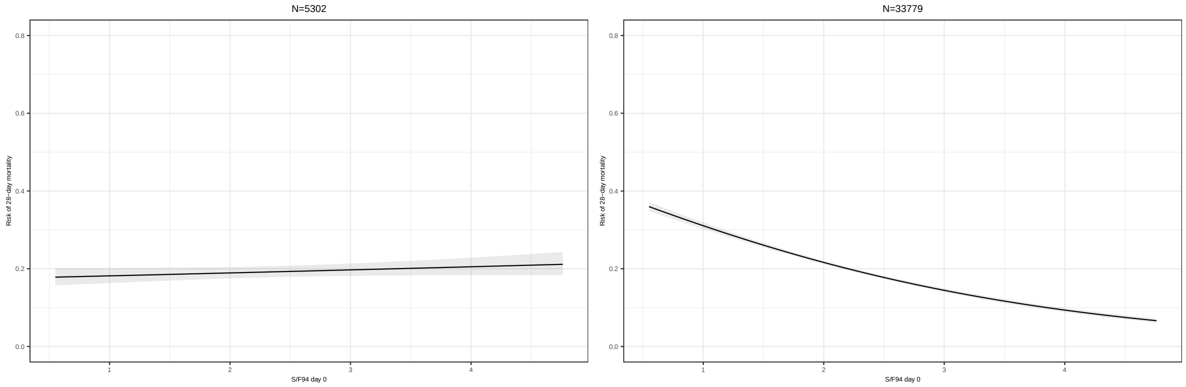

(a) Multivariate regression model

(b) Univariate regression model

Supplementary Figure 4: Results of regression model (including 95% confidence interval) for S/F_{94}, using mortality at day 28 as the outcome, in (a) multivariable model including both day 0 and day 5 S/F_{94} and (b) a univariate model with day 0 S/F_{94} as the sole predictor variable. The univariate model shows a clear and expected association between mortality at day 28 and S/F_{94} on day 0.

to 1.29 in the main analysis). The average change in S/F_{94} associated with a 15% reduction in risk of mortality was the same in main analysis and this sensitivity analysis (0.18), and correlation between S/F_{94} on day 0 and day 5 was almost identical in both analyses (0.31 in the main analysis compared to 0.32 in this sensitivity analysis). As a consequence of the lower mean and standard deviation of S/F_{94}, the sample size required decreases from 1,444 in the main analysis, to 884 in this sensitivity analysis.

Inclusion criteria

We compared the effect different inclusion criteria had on required sample sizes (Supplementary Tables 2, 3 and 4). In general, as expected, the more broad the inclusion criteria (e.g. only age limits), the larger the required sample size; and the more specific the inclusion criteria, the smaller the require sample size. However, it is important to note that using more stringent inclusion criteria increases the burden associated with screening patients for inclusion in a study, which may substantially decrease real-world recruitment.

Selection criteria	$S_pO_2 \leq 0.94$ or F_IO_2= 0.21	S_pO_2> 0.94	Day 0 and ($S_pO_2 \leq 0.94$ or F_IO_2= 0.21)	Day 0 and S_pO_2> 0.94
Number of entries	192,583	55,765	114,668	27,367
Unique subjects	129,049	39,457	114,688	27,367

Selection criteria	$S_pO_2 \leq 0.94$ or F_IO_2= 0.21	S_pO_2> 0.94	Day 0 and ($S_pO_2 \leq 0.94$ or F_IO_2= 0.21)	Day 0 and S_pO_2> 0.94
Age, years	71 (57-82)	69 (55-82)	72 (58-83)	70 (55-82)
Male	111,244 (58%)	32,022 (58%)	63,127 (55%)	15,351 (56%)
Respiratory rate, per minute	20 (18-25)	22 (18-26)	20 (18-25)	22 (18-25)
Temperature, Celcius	37.2 (36.8-38.0)	37.1 (36.7-37.8)	37.3 (36.8-38.1)	37.3 (36.8-38.1)
Systolic blood pressure, mmHg	116 (103-138)	120 (105-140)	117 (104-138)	121 (106-142)
Diastolic blood pressure, mmHg	66 (57-77)	69 (59-77)	68 (58-79)	70 (60-81)
CRP, mg/L	85 (39-159)	84 (39-152)	78 (34-145)	84 (40-149)
BUN, mmol/L	7.0 (4.8-10.9)	6.7 (4.6-10.5)	6.7 (4.6-10.3)	6.5 (4.5-10.1)
Creatinine, umol/L	86 (68-120)	84 (67-119)	88 (70-119)	86 (70-116)
Urine production, ml/24h	1,001 (400-1839)	1,020 (450-1890)	500 (300-950)	550 (300-1,000)
Glasgow Coma Scale	15 (15-15)	15 (15-15)	15 (15-15)	15 (15-15)
Clinical frailty	4 (2-6)	4 (2-6)	4 (3-6)	4 (2-6)
Onset to admission, days	4 (0-8)	4 (0-8)	3 (0-7)	4 (0-8)
Infiltrates on X-ray preent	52,001 (67%)	14,525 (69%)	41,944 (63%)	12,147 (67%)
No respiratory support (WHO 4)	71,633 (37%)	0 (0%)	11,187 (23%)	0 (0%)
Nasal oxygen (WHO 5)	53,610 (28%)	23,220 (42%)	19,904 (41%)	10,102 (64%)
Non invasive ventilation (WHO 6)	46,473 (24%)	11,764 (21%)	15,370 (32%)	5,148 (32%)
IMV: SF >2.0 (WHO 7)	3,063 (2%)	1,789 (3%)	249 (<1%)	172 (1%)

Selection criteria	$S_pO_2 \leq 0.94$ or F_IO_2= 0.21	S_pO_2> 0.94	Day 0 and ($S_pO_2 \leq 0.94$ or F_IO_2= 0.21)	Day 0 and S_pO_2> 0.94
IMV: SF ≤ 2.0 or vasopressor (WHO 8)	8,782 (5%)	1.900 (3%)	1,174 (2%)	345 (2%)
IMV: SF 2.0 and vasopressor, ECMO or dialysis (WHO 9)	3,004 (2%)	784 (1%)	401 (<1%)	151 (1%)
Death (WHO 10)	30,225 (23%)	8,289 (21%)	26,017 (23%)	5,924 (22%)
Discharged alive (including transfer to another facility)	98,824 (77%)	31,168 (79%)	88,651 (77%)	21,443 (78%)

Supplementary Table 1: Characteristics of patient populations meeting criteria for S/F_{94} measurement in ISARIC4C data. Continuous values are summarised as median (inter quartile range). Categorical values are summarised as frequency (percentage). Subjects meeting the criteria for S/F_{94} ($S_pO_2 \leq 0.94$ or $F_IO_2 = 0.21$) were compared to patients not meeting the S/F_{94} criteria. Comparison was done both for all entries during admission and day 0. CRP = C-reactive protein, BUN= blood urea nitrogen.

Measure	Distribution/ Event rate	Estimated treatment effect	Total n (= 80% 2p = 0.05)
S/F_{94} day 5	Mean = 2.38<br>SD = 1.31<br>ρ vs Day 0 = 0.42	$\Delta S/F_{94}$: 0.177	1,430
S/F_{94} day 8	Mean = 2.74<br>SD = 1.53<br>ρ vs Day 0 = 0.39	$\Delta S/F_{94}$: 0.153	2.696
WHO day 5	4: 1,979/8,270<br>5: 1,861/8,270<br>6: 2,270/8,270<br>7: 301/8,270<br>8: 980/8,270<br>9: 313/8,270<br>10: 566/8,270	Proportional odds ratio: 0.84	3,293

Measure	Distribution/ Event rate	Estimated treatment effect	Total n (= 80% 2p = 0.05)
WHO day 8	4: 2,478/6,916<br>5: 1,206/6,916<br>6: 1,196/6,916<br>7: 361/6,916<br>8: 812/6,916<br>9: 261/6,916<br>10: 602/6,916	Proportional odds ratio: 0.84	3,227
1-level sustained improvement	12,057/26,854 (44.9%)	Risk ratio: 1.03	6,544
2-level sustained improvement	4,914/26,854 (18.3%)	Risk ratio: 1.04	3,722
28-day mortality	7,583/36,559 (20.7%)	Risk ratio: 0.85	5,154

Supplementary Table 2: Comparison of outcome measures among 36,559 hospitalised patients aged 20-75, who required supplemental oxygen on the day of admission. The estimated treatment effect is for a 15% relative reduction in mortality. For S/F_{94}, the mean value and standard deviation (SD) and the correlation with S/F_{94} on day 0 are reported for both day 5 and day 8. For the WHO ordinal severity scale, the number of people in each group between 4 (hospitalised, no oxygen support) and 10 (dead) are reported. For 1- and 2-level sustained improvement, the number of people reaching sustained improvement is reported. For 28-day mortality, the proportion of patients who died at day 28 is reported. Sample sizes shows the total number of subjects needed in both arms using a 1:1 allocation.

Measure	Distribution/ Event rate	Estimated treatment effect	Total n (= 80% 2p = 0.05)
S/F_{94} day 5	Mean = 2.83<br>SD = 1.47<br>ρ vs Day 0 = 0.56	$\Delta S/F_{94}$: 0.173	1,552
S/F_{94} day 8	Mean = 3.15<br>SD = 1.57<br>ρ vs Day 0 = 0.46	$\Delta S/F_{94}$: 0.150	2.692

Measure	Distribution/ Event rate	Estimated treatment effect	Total n (= 80% 2p = 0.05)
WHO day 5	4: 5,242/14,004<br>5: 2,903/14,004<br>6: 3,091/14,004<br>7: 385/14,004<br>8: 1,198/14,004<br>9: 438/14,004<br>10: 747/14,004	Proportional odds ratio: 0.84	3,504
WHO day 8	4: 5,562/11,881<br>5: 1,880/11,881<br>6: 1,688/11,881<br>7: 455/11,881<br>8: 1,037/11,881<br>9: 370/11,881<br>10: 889/11,881	Proportional odds ratio: 0.84	3,527
1-level sustained improvement	14,556/47,260 (30.8%)	Risk ratio: 1.03	6,061
2-level sustained improvement	5,860/47,260 (12.4%)	Risk ratio: 1.04	2,959
28-day mortality	10,811/69,464 (15.6%)	Risk ratio: 0.85	7,267

Supplementary Table 3: Comparison of outcome measures among 69,464 hospitalised patients aged 20-75. The estimated treatment effect is for a 15% relative reduction in mortality. For S/F_{94}, the mean, standard deviation (SD) and correlation with S/F_{94} on day 0 are reported for both day 5 and day 8. For the WHO ordinal severity scale, the number of people in each category between 4 (hospitalised, no oxygen support) and 10 (dead) are reported. For 1- and 2-level sustained improvement, the number of people reaching sustained improvement is reported. For 28-day mortality, the proportion of patients who died at day 28 is reported. Sample sizes shows the total number of subjects needed in both arms using a 1:1 allocation.

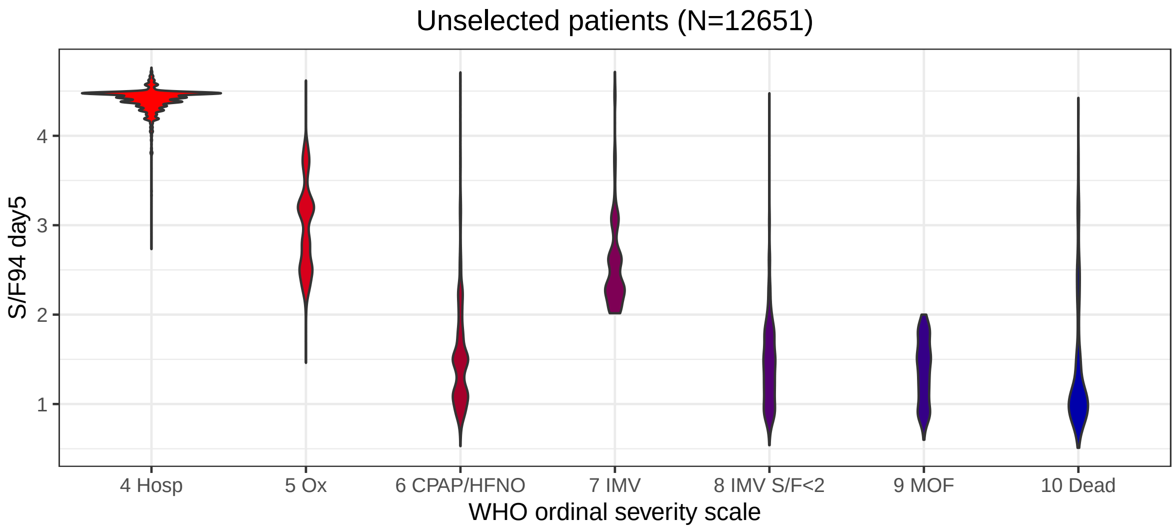

(a) S/F_{94} on Day 5 by WHO ordinal scale

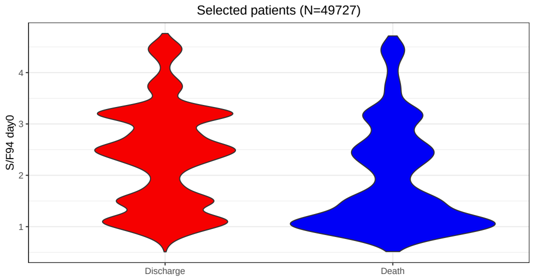

(b) S/F_{94} on Day 0

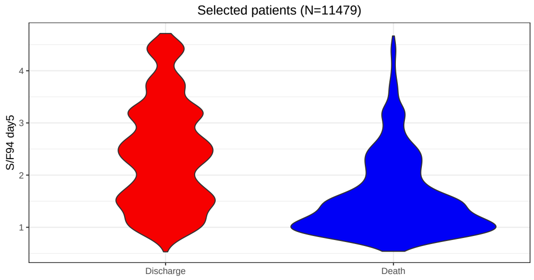

(c) S/F_{94} on Day 5

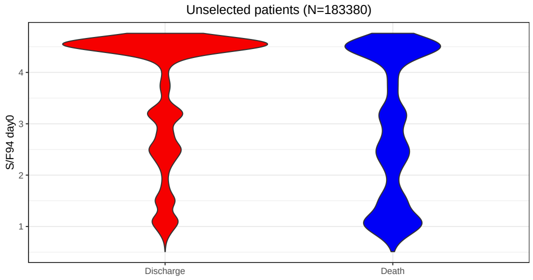

(d) S/F_{94} on Day 0

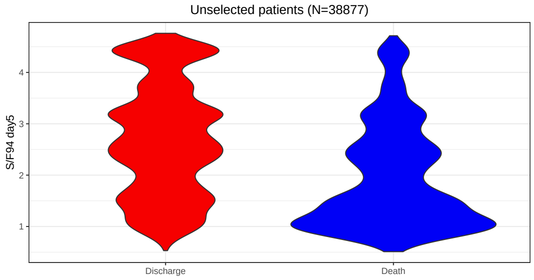

(e) S/F_{94} on Day 5

Supplementary Figure 5: Relationship between (a) Distribution of S/F_{94} values day 5 compared with WHO ordinal scale value at the same time point. For some patients who died on day 5, S/F_{94} values were available. (b) 28-day outcome and S/F_{94} on day 0 and (c) 28-day outcome and S/F_{94} on day 5 among selected patients meeting inclusion criteria (age 20-75; oxygen therapy in first 3 days) (d) 28-day outcome and S/F_{94} on day 0 and (e) 28-day outcome and S/F_{94} on day 5 among unselected patients. Hosp = hospitalised, no oxygen support; Ox = Hospitalised, oxygen by mask or nasal prongs; CPAP/HFNO = Hospitalised, oxygen by continuous positive airway pressure; high-flow nasal oxygen or non-invasive ventilation; IMV = Intubation and mechanical ventilation; IMV $S/F \leq 2$ = Mechanical ventilation; $S/F \leq 2$ or vasopressors; MOF = Multi-organ failure & mechanical ventilation & $S/F_{94} \leq 2$ & ECMO or renal replacement therapy.

Measure	Distribution/ Event rate	Estimated treatment effect	Total n (= 80% 2p = 0.05)
S/F_{94} day 5	Mean = 2.39<br>SD = 1.29<br>ρ vs Day 0 = 0.31	$\Delta S/F_{94}$: 0.180	1,444
S/F_{94} day 8	Mean = 2.73<br>SD = 1.51<br>ρ vs Day 0 = 0.28	$\Delta S/F_{94}$: 0.158	2.684
WHO day 5	4: 2,275/9,998<br>5: 2,433/9,998<br>6: 2,736/9,998<br>7: 363/9,998<br>8: 1,145/9,998<br>9: 418/9,998<br>10: 628/9,998	Proportional odds ratio: 0.84	3,331
WHO day 8	4: 2,801/8,207<br>5: 1,525/8,207<br>6: 1,445/8,207<br>7: 429/8,207<br>8: 968/8,207<br>9: 343/8,207<br>10: 696/8,207	Proportional odds ratio: 0.84	3,222
1-level sustained improvement	13,437/30,060 (44.7%)	Risk ratio:: 1.03	6,756
2-level sustained improvement	5,411/30,060 (18.0%)	Risk ratio: 1.04	3,808
28-day mortality	8,262/39,765 (20.8%)	Risk ratio: 0.85	5,143

Supplementary Table 4: Comparison of outcome measures among 39,765 hospitalised patients aged 20-75, who required supplemental oxygen in the first 3 days in hospital. The estimated treatment effect is for a 15% relative reduction in mortality
