# Supplementary material for: Evaluation of pragmatic oxygenation measurement as a proxy for Covid-19 severity
Source: Nat Commun. 2023 Nov 15;14:7374. doi: 10.1038/s41467-023-42205-6 (PMC10651917; doi:10.1038/s41467-023-42205-6)
Supplement: Supplementary file 3 — Reporting Summary [file 41467_2023_42205_MOESM3_ESM.pdf]

nature portfolio

Corresponding author(s): J. Kenneth Baillie

Last updated by author(s): Sep 26, 2023

Reporting Summary

Nature Portfolio wishes to improve the reproducibility of the work that we publish. This form provides structure for consistency and transparency in reporting. For further information on Nature Portfolio policies, see our Editorial Policies and the Editorial Policy Checklist.

Statistics

For all statistical analyses, confirm that the following items are present in the figure legend, table legend, main text, or Methods section.

n/a	Confirmed	
☐	☒	The exact sample size (*n*) for each experimental group/condition, given as a discrete number and unit of measurement
☐	☒	A statement on whether measurements were taken from distinct samples or whether the same sample was measured repeatedly
☐	☒	The statistical test(s) used AND whether they are one- or two-sided<br>*Only common tests should be described solely by name; describe more complex techniques in the Methods section.*
☐	☒	A description of all covariates tested
☐	☒	A description of any assumptions or corrections, such as tests of normality and adjustment for multiple comparisons
☐	☒	A full description of the statistical parameters including central tendency (e.g. means) or other basic estimates (e.g. regression coefficient) AND variation (e.g. standard deviation) or associated estimates of uncertainty (e.g. confidence intervals)
☐	☒	For null hypothesis testing, the test statistic (e.g. *F*, *t*, *r*) with confidence intervals, effect sizes, degrees of freedom and *P* value noted<br>*Give P values as exact values whenever suitable.*
☒	☐	For Bayesian analysis, information on the choice of priors and Markov chain Monte Carlo settings
☒	☐	For hierarchical and complex designs, identification of the appropriate level for tests and full reporting of outcomes
☒	☐	Estimates of effect sizes (e.g. Cohen's *d*, Pearson's *r*), indicating how they were calculated

Our web collection on statistics for biologists contains articles on many of the points above.

Software and code

Policy information about availability of computer code

Data collection	No software was used to collect the data
Data analysis	The code used to perform the analyses can be found on a public github repository (https://github.com/baillielab/SF94)

For manuscripts utilizing custom algorithms or software that are central to the research but not yet described in published literature, software must be made available to editors and reviewers. We strongly encourage code deposition in a community repository (e.g. GitHub). See the Nature Portfolio guidelines for submitting code & software for further information.

Data

Policy information about availability of data

All manuscripts must include a data availability statement. This statement should provide the following information, where applicable:

- Accession codes, unique identifiers, or web links for publicly available datasets
- A description of any restrictions on data availability
- For clinical datasets or third party data, please ensure that the statement adheres to our policy

Source data are provided for figure 1 and supplementary figure 1 and 2. The dataset used and analysed in this study contains clinical data about individuals and is available after a data access request. Data access request and details on the procedure can be found at https://odap.ac.uk/researchers . Data access requests will be reviewed on the basis of scientific merit and validity, the proposed timeline, ethical considerations and the available resources. Access requests can be send to odap@ed.ac.uk. A reply to a data access request will be provided within six weeks from the date of the request. Depending on the requested data, there may be additional steps before data can be published, such as agreement from all contributors. For details, please see https://odap.ac.uk/researchers . All data supporting

the findings in this manuscript are present in the main text, supplementary material, the source data and from the corresponding author upon request. A synthetically generated dataset, containing the same key properties as the original dataset is available for sample size calculations on https://isaric4c.net/endpoints

Research involving human participants, their data, or biological material

Policy information about studies with human participants or human data. See also policy information about sex, gender (identity/presentation), and sexual orientation and race, ethnicity and racism.

Reporting on sex and gender	Only the term sex is used in our manuscript, as no data on gender was available. Sex was not considered in the study design. Data on sex was collected based on electronic health records.
Reporting on race, ethnicity, or other socially relevant groupings	No data on race or ethnicity was available in our dataset.
Population characteristics	Covariates used in this study are age and sex. Patients <18 years old were exluded, but no other restrictions on age or sex were present.
Recruitment	Patients hospitalised with SARS-CoV-2 infection in England, Scotland or Wales, were eligible for inclusion in this study
Ethics oversight	Ethical approval was given by the South Central-Oxford C Research Ethics Committee in England (13/SC/0149), the Scotland A Research Ethics Committee (20/SS/0028), and the WHO Ethics Review Committee (RPC571 and RPC572, April 2013).<br><br>In England and Wales, consent was not required for the collection of depersonalised routine healthcare research data. In Scotland, a waiver for consent was given by the Public Benefit and Privacy Panel

Note that full information on the approval of the study protocol must also be provided in the manuscript.

Field-specific reporting

Please select the one below that is the best fit for your research. If you are not sure, read the appropriate sections before making your selection.

☒ Life sciences ☐ Behavioural & social sciences ☐ Ecological, evolutionary & environmental sciences

For a reference copy of the document with all sections, see nature.com/documents/nr-reporting-summary-flat.pdf

Life sciences study design

All studies must disclose on these points even when the disclosure is negative.

Sample size	No sample size calculation was performed in advance for our study. The study was carried out prospectively with all data that was available to us in order to investigate if our research question could be usefully answered.
Data exclusions	Patients under 18 years old were excluded from the analysis
Replication	We repeated our analysis using different inclusion criteria to confirm our findings. We used 3 different sets of inclusion criteria, and repeated our analysis in all three sets.
Randomization	As the data was collected as part of an observational study, we did not randomise participants. Patients the the companion paper on the RECOVERY trial were randomised - see manuscript for details
Blinding	As the data was collected as part of an observational study, blinding is not relevant

Reporting for specific materials, systems and methods

We require information from authors about some types of materials, experimental systems and methods used in many studies. Here, indicate whether each material, system or method listed is relevant to your study. If you are not sure if a list item applies to your research, read the appropriate section before selecting a response.

Materials & experimental systems

n/a	Involved in the study
☒	☐ Antibodies
☒	☐ Eukaryotic cell lines
☒	☐ Palaeontology and archaeology
☒	☐ Animals and other organisms
☒	☐ Clinical data
☒	☐ Dual use research of concern
☒	☐ Plants

Methods

n/a	Involved in the study
☒	☐ ChIP-seq
☒	☐ Flow cytometry
☒	☐ MRI-based neuroimaging
